# Oral Leptin Treatment in Suckling Rats Ameliorates Detrimental Effects in Hypothalamic Structure and Function Caused by Maternal Caloric Restriction during Gestation

**DOI:** 10.1371/journal.pone.0081906

**Published:** 2013-11-28

**Authors:** Jadwiga Konieczna, Ana Paula García, Juana Sánchez, Mariona Palou, Andreu Palou, Catalina Picó

**Affiliations:** Laboratory of Molecular Biology, Nutrition and Biotechnology (Nutrigenomics), University of the Balearic Islands (UIB) and CIBER Fisiopatología de la Obesidad y Nutrición (CIBEROBN), Palma de Mallorca, Spain; Pennington Biomedical Research Center, United States of America

## Abstract

A poor prenatal environment brings about perturbations in leptin surge and hypothalamic circuitry that program impaired ability to regulate energy homeostasis in adulthood. Here, using a rat model of moderate maternal caloric restriction during gestation, we aimed to investigate whether leptin supplementation with physiological doses throughout lactation is able to ameliorate the adverse developmental malprogramming effects exerted in offspring hypothalamus structure and function. Three groups of male and female rats were studied: the offspring of *ad libitum* fed dams (controls), the offspring of 20% calorie restricted dams during the first part of pregnancy (CR), and CR rats supplemented with physiological doses of leptin throughout lactation (CR-Leptin). Animals were sacrificed on postnatal day 25. Morphometric and immunohistochemical studies on arcuate (ARC) and paraventicular (PVN) nucleus were performed and hypothalamic expression levels of selected genes were determined. In CR males, leptin treatment restored, at least in part, the number of immunoreactive neuropeptide Y (NPY^+^) cells in ARC, the total number of cells in PVN, hypothalamic NPY, cocaine- and amphetamine-regulated transcript (CART) and suppressor of cytokine signalling-3 (SOCS-3) mRNA levels, and plasma leptin levels, which were decreased in CR animals. CR-Leptin males showed higher hypothalamic long-form leptin receptor (ObRb) mRNA levels, compared to control and CR animals. In CR females, leptin treatment reverted the increased number of cells in ARC and cell density in ARC and PVN, and reduced hypothalamic SOCS-3 mRNA expression to levels similar to controls. Leptin treatment also reverted the increased relative area of NPY^+^ fibers in the PVN occurring in CR animals. In conclusion, leptin supplementation throughout lactation is able to revert, at least partly, most of the developmental effects on hypothalamic structure and function caused by moderate maternal caloric restriction during gestation, and hence making this metabolic malprogramming reversible to some extent.

## Introduction

Obesity is multifactorial in origin, but is mostly attributed to the interaction between environmental conditions and genetic predisposition [1]. However, it is becoming increasingly apparent that nutritional factors during early stages of development can lead to permanent programming of central and peripheral systems that regulate energy balance [2–7]. Obesity is associated with severe morbidity and is increasing in prevalence, thus determination of strategies enabling its prevention starting in the early stages of life, as well as the potentialities to reverse early programmed effects of obesity-related metabolic disorders, become of crucial relevance. 

A considerable number of studies have addressed the lasting consequences of an adverse prenatal environment on health outcomes in the offspring. In this view, maternal prenatal malnutrition, as well as low birth weight, have been described to be associated with obesity and metabolic syndrome in adulthood [8–11]. The first, renowned evidence in scientific literature supporting this proposal comes from an epidemiological study on the consequences of the Dutch Hunger Winter famine, near the end of World War II. This study demonstrated that males born to mothers who underwent malnutrition during the first 2 trimesters of gestation were more likely to be obese in adult life [8]. In current day, the presence of maternal undernutrition during gestation does not appear to account for the increasing prevalence of obesity in children and adults in developed societies, but this phenomenon could better explain the increasing prevalence of obesity among people in developing countries, which undergo the transition from chronic malnutrition to adequate or excessive nutrition [12,13]. Animal studies leading to a better understanding of this association have pointed out that maternal food restriction during pregnancy is a risk factor increasing vulnerability to later obesogenic environmental stimuli [5,6,14]. In particular, moderate caloric restriction during the first half of gestation in rats has been described to program the offspring for greater food intake as well as for insulin and leptin resistance, which results in higher body weight and body fat content in males but not in females [6,15]. Despite these observations, elucidation of the mechanisms responsible for these ‘developmental origins of health and disease’ is a topic of great concern and still remains unclear.

There is emerging evidence showing that specific hypothalamic areas that regulate food intake and energy expenditure may be particularly susceptible to permanent programming by the early nutritional and hormonal environment, which could influence the capacity to regulate energy homeostasis in adulthood. In this sense, we and others have evidenced that nutritional manipulations during perinatal period, such as protein restriction [16,17] or caloric restriction [18–20], modify hypothalamic structure and function. In particular, perinatal 50% food restriction reduces nerve fibers immunoreactive to beta-endorphin (a product of POMC) projecting from the arcuate nucleus (ARC) to the paraventicular nucleus (PVN) in neonate rats [18]. In turn, more moderate maternal caloric restriction (20%) during pregnancy also perturbed hypothalamic ARC structure in weaned rats, by decreasing the presence of total number of cells, and particularly NPY-neurons [20].

Leptin, an adipocyte-derived hormone, plays a central role regulating energy balance. It acts particularly at the hypothalamic ARC to attenuate hunger, by inhibiting orexigenic neuropeptides (such as NPY and AgRP) and stimulating anorexigenic ones (such as POMC and CART), as well as increasing energy expenditure [21,22]. In addition to its role in the regulation of energy homeostasis in adults, during early steps of development, leptin has been shown to play a crucial neurotrophic role in programming hypothalamic circuit formation, particularly nerves projections from ARC to the other hypothalamic areas [23]. Neurodevelopment action of leptin appears to be restricted to the second week of life, which is coincident with a rise in circulating leptin levels, the so-called leptin surge [23,24]. Severe perinatal maternal food restriction (50%) [18], or even moderate maternal caloric restriction (20%) during the first 12 days of gestation, which has been shown to determine alterations in hypothalamic circuitry [15], has been found to be associated with a drastic reduction or even absence of postnatal leptin surge in the offspring. In this regard, studies in rats have evidenced that leptin supplied by milk or as a water solution during the suckling period can be absorbed by the immature stomach [25–27] and be transferred to the bloodstream [25,27]. Therefore, maternal milk may substantially contribute to circulating leptin in neonate rats, at a time when the adipose tissue is still immature [28]. Hence, considering the neurotrophic role of leptin, it could be speculated that leptin supplementation during a critical window of developmental plasticity may have the ability to reverse some of the neuroanatomical defects and other features of the obese phenotype associated with absence or alterations in neonatal leptin surge [23,24]. Interestingly, daily intraperitoneal injections of pharmacological doses of leptin (10 mg/kg, between postnatal day 4 and 12) in leptin deficient mice (ob/ob) has been shown to rescue the development of disrupted ARC projections [24]. Vickers et al. [29] also evidenced that daily leptin subcutaneous injection (2.5 µg/g/d) from postnatal day 3-13 in female pups born from 30% calorie-restricted dams during gestation, normalized calorie intake, locomotor activity, body weight, fat mass, and fasting plasma glucose, insulin and leptin concentrations in adulthood. The same treatment with pharmacological doses of leptin in early undernourished male pups conferred protection against obesity development, but only when animals were under HF diet [30]. Notably, we have also described that neonate male rats born from adequately nourished dams, and orally treated with physiological amounts of leptin throughout the suckling period are more resistant to age-related increases in body weight and diet-induced weight gain [31], displaying improved insulin and leptin sensitivity [32,33], and showing lower preference for fat-rich food in adulthood [32]. Overall, these results point out the essential role of leptin during lactation in imprinting healthier metabolic responses in later life [28]. However, no studies have been conducted so far considering the ability of oral leptin supplementation at physiological doses during lactation to reverse early malprogramming effects in hypothalamus associated to poor prenatal conditions. 

In the present study, we used an experimental rat model of moderate (20%) maternal caloric restriction during pregnancy, which is known to be associated with alterations in hypothalamic circuitry that program a higher propensity to develop obesity in the offspring, particularly in males [6], to investigate whether oral supplementation with physiological doses of leptin throughout lactation is able to ameliorate or normalize developmental malprogramming of hypothalamus, which may be responsible, at least in part, for the adverse health outcomes later in life.

## Materials and Methods

### Animals and Experimental Design

The animal protocol followed in this study was reviewed and approved by the Bioethical Committee of the University of the Balearic Islands (Resolution Number 1798. February 18th, 2009) and guidelines for the use and care of laboratory animals of the University were followed.

The study was conducted on male and female Wistar rat pups from 17 different litters following the protocol during pregnancy and lactation as is described below. Animals were housed under standard conditions, that is, controlled temperature (22 C), the normal 12-h light and 12-h dark cycle, free access to tap water and a standard laboratory rodent chow diet (3.3 kcal/g, with 8% calories from fat; Panlab, Barcelona, Spain), unless specified. Virgin female Wistar rats (body weight 217 g - 244 g) were mated with male rats (Charles River Laboratories, Barcelona, Spain). Day of conception (day 0 of pregnancy) was determined by examination of vaginal smears for the presence of sperm. Pregnant rats were divided into two groups: control dams (n=7 animals) fed *ad libitum* with standard chow diet, and calorie restricted dams (CR-dams) (n=10 animals) fed with 20% caloric restriction from day 1 to day 12 of gestation, as previously described [6]. After the calorie restriction period, all dams were fed *ad libitum*, and food intake was measured. 

On day 1 after delivery, excess pups in each litter were removed to keep 10 pups per dam (five males and five females, when possible). Pups of both sexes born from CR-dams were randomly assigned into two groups: CR and CR-Leptin. CR-Leptin animals were supplemented, each day throughout lactation with an oral solution of recombinant murine leptin (PeproTech, London, UK) dissolved in water by using a pipette. The amount of leptin given to animals was progressively increased from 1 ng of leptin on day 1, to 43.8 ng of leptin on day 20 of life, as previously described [31]. CR pups and the offspring of control dams (controls) received the same volume of the vehicle (water).

Pups were weaned at 21 days of life, and 35 pups from control group (18 males and 17 females), 34 from CR group (17 males and 17 females), and 33 from CR-Leptin group (17 males and 16 females) were housed in groups of two animals, and fed on a standard chow diet. Body length (from the tip of the nose to the anus) and body fat content (by EchoMRI-700^TM^, Echo Medical Systems, LLC., TX, USA) were measured in all the animals when animals were 25 days old. Body weight and food intake were recorded from weaning until the age of 25 days.

On day 25, pups were sacrificed by decapitation, during the first 2 h of the beginning of the light cycle, under fed conditions. Some of these animals (n = 10–11, per group) were used for gene expression analysis and the others (n = 6-8, per group) to perform morphometric and immunohistochemical analysis. Animals used for the different analysis were from at least six different litters. 

Blood samples were collected in heparinized containers, then centrifuged at 1000 x *g* for 10 min to obtain the plasma, and stored at −20 C until analysis. For gene expression studies, the hypothalamus was rapidly removed, immediately frozen in liquid nitrogen and stored at −80 C until RNA analysis. For morphometric analysis, brain samples were fixed by immersion in 4% paraformaldehyde in 0.1 M phosphate buffer (pH = 7.4) at 4 C for 24 h, then washed and stored in 0.1 M phosphate buffer (pH = 7.4) until posterior analysis.

### Quantification of Glucose, Insulin and Leptin Concentration

Blood glucose concentration was measured by Accu-Chek Glucometer (Roche Diagnostics, Barcelona, Spain). Plasma insulin concentration was determined using ultrasensitive rat insulin enzyme-linked immunosorbent assay (ELISA) kit (Mercodia AB, Uppsala, Sweden). Leptin concentration in plasma and in stomach homogenates was measured using ELISA kit Quantikine^TM^ Mouse Leptin Inmunoassay (R&D Systems, Minneapolis, MN, USA) as previously described [27]. 

### RNA Extraction

Total RNA was extracted from hypothalamus and stomach using TRIpure Reagent (Roche Diagnostic Gmbh, Mannheim, Germany), in accordance to the manufacturer´s instructions. RNA yield was quantified on the NanoDrop ND-1000 spectrophotometer (NadroDrop Technologies, Wilmington, DE, USA) and its integrity confirmed using 1% agarose gel electrophoresis. 

### Real-time Quantitative PCR Analysis

Real-time polymerase chain reaction was used to measure mRNA expression levels of Agouti-related peptide (AgRP), cocaine- and amphetamine-regulated transcript (CART), neuropeptide Y (NPY), long-form leptin receptor (ObRb), proopiomelanocortin (POMC), and suppressor of cytokine signalling-3 (SOCS-3) in hypothalamus and leptin mRNA levels in stomach. qRT-PCR analysis was performed as previously described [15]. Primer sequences and products for the different genes are described in Table 1. All primers were purchased from Sigma Genosys (Sigma Aldrich Quımica SA, Madrid, Spain). In order to verify the purity of the products, a melting curve was produced after each run. The values for the threshold (Ct) were calculated by the instrument’s software (StepOne Software v2.2.2), and the relative expression of each mRNA was calculated as a percentage of male control rats, using the 2^-∆∆Ct^ method [34] with β-actin and rho gdp dissociation inhibitor alpha (GDI) (in hypothalamus) and 18S ribosomal (in stomach) as reference genes.

**Table 1:** Nucleotide sequences of primers and amplicon size used for qRT-PCR.

**Gene**	**Forward primer (5' to 3')**	**Reverse primer (5' to 3')**	**Amplicon (bp)**
18S	CGCGGTTCTATTTTGTTGGT	AGTCGGCATCGTTTATGGTC	219
β-actin	TACAGCTTCACCACCACAGC	TCTCCAGGGAGGAAGAGGAT	120
AgRP	AGAGTTCTCAGGTCTAAGTCT	CTTGAAGAAGCGGCAGTAGCACGT	210
CART	AGAAGAAGTACGGCCAAGTCC	CACACAGCTTCCCGATCC	84
GDI	CCGCACAAGGCAAATACATC	GACTCTCTGAACCGTCATCAA	210
Leptin	TTCACACACGCAGTCGGTAT	AGGTCTCGCAGGTTCTCCAG	186
NPY	TGGACTGACCCTCGCTCTAT	GTGTCTCAGGGCTGGATCTC	188
ObRb	AGCCAAACAAAAGCACCATT	TCCTGAGCCATCCAGTCTCT	174
POMC	CCTGTGAAGGTGTACCCCAATGTC	CACGTTCTTGATGATGGCGTTC	266
SOCS-3	ACTGAGCCGACCTCTCTCCT	CCCCTCTGACCCTTTCTTTG	172

Abbreviations: AgRP, Agouti-related peptide; CART, cocaine- and amphetamine-regulated transcript; GDI, rho gdp dissociation inhibitor alpha; NPY, neuropeptide Y; ObRb, long-form leptin receptor; POMC, proopiomelanocortin; SOCS-3, suppressor of cytokine signalling-3. Thermal cycling conditions for all genes, with the exception of leptin were as follows: 10 min at 95°C followed by a total of 40 two-temperature cycles (15 s at 95°C and 1 min at 60°C). For leptin, conditions were as follows: 7 min at 95°C followed by a total of 50 three-temperature cycles (15 s at 95°C, 20 s at 60°C and 40 s at 72°C).

### Morphometric and Immunohistochemical Analysis

In the fixed brains a coronal block containing the hypothalamus was cut, dehydrated in graded series of ethanol, cleared in xylene and embedded in paraffin. Coronal sections (5 μm thick) from the hypothalamus were cut using a microtome and mounted on Super-Frost/Plus slides.

Immunohistochemical demonstration of NPY in ARC and PVN was performed with the avidin-biotin peroxidise (ABC) method [35,36]. Sections were incubated sequentially at room temperature in the following solutions: 0.3% hydrogen peroxide in methanol for 10 min to block endogenous peroxidase; Citrate-based solution (pH 6) in microwave oven for 15 min and 20 min on ice for antigen retrieval; 2% goat normal serum in phosphate buffered saline (PBS) (pH 7.4-7.6) 0.1% Triton X-100 for 20 min to reduce non-specific background staining prior to incubation with primary antibody (polyclonal anti-NPY antibody produced in rabbit, N9528, Sigma-Aldrich, 1:800 in PBS 0.1% Triton X-100 with 1% BSA for 1 h and 15 min at 37 C); biotinylated goat antirabbit IgG (Vector Laboratories, Burlingame, CA) 1:200 in PBS 0.1% Triton X-100 with 1% BSA for 1 h at room temperature; peroxidase-labeled ABC reagent (Vectastain ABC kit, Vector) in PBS for 30 min at room temperature and Fast 3,3-diaminobezidine tablet, DAB (Sigma, St. Louis, MO,USA) in PBS 0.1% Triton X-100 for 3 min in dark room for enzymatic development of peroxidise. Subsequently, slides were washed with deionized water, dehydrated with increasing concentrations of ethanol and xylene, mounted with Eukitt (Panreac Quimica SA) and cover-slipped. Negative controls were performed by omission of primary antibody. Measurement of the number of immunoreactive NPY (NPY^+^) neurons in ARC and the area occupied by NPY^+^ fibers in PVN, were performed in 3 digitalized images/animal from the ARC (−2.3 to −3.3 mm posterior to Bregma) and from the PVN (−1.6 to −1.88 mm posterior to Bregma) according to published coordinates [35] and with the help of hematoxylin/eosin staining. 

The area occupied and the number of hematoxylin/eosin-stained cells were also measured. Capture of images (at a x10 magnification) and analysis were performed using AxioVision40V 4.6.3.0. Software (Carl Zeiss, Imaging Solutios GmbH, Germany). Image analysis from all groups was examined by two independent researchers in a blind fashion. 

### Statistical Analysis

Data are reported as mean ± standard error (SEM). Two-way ANOVA with sex and group factors showed that male and female pups respond in a different manner to the treatments used. Therefore, one-way ANOVA was performed separately for each sex, to study individual differences between groups (controls, CR, CR-Leptin), followed by least significance difference (LSD) *post hoc* test. The data were confirmed for equality of variances by Levene's test (p < 0.05). Single comparisons between groups were assessed by Student’s *t* test. Threshold of significance was set at p < 0.05. All the analyses were performed with SPSS Statistics 19.0 (SPSS, Chicago, IL). 

## Results

### Body weight gain and energy intake in dams

Body weight gain and cumulative energy intake of dams during different periods of gestation and lactation are summarized in Table 2. 20% food restriction conducted at the beginning of gestation (day 1 to 12) resulted in lower weight gain of CR-dams and, consequently, these animals showed lower body weight at the end of the restriction period (day 12) with respect to their controls (controls-dams: 286 ± 3 g and CR-dams: 251 ± 3 g) (p < 0.05; Student’s *t* test). During the second part of gestation (day 13 to 20), when all dams were allowed to eat *ad libitum*, CR-dams gradually regained body weight of controls, but at day 20 of gestation they still showed a slight tendency to lower body weight compared to their controls (controls-dams: 359 ± 7 g and CR-dams: 339 ± 8 g) (p = 0.09; Student’s *t* test). During the lactation period, although both groups of dams consumed a similar amount of food, CR-dams gained significantly more body weight than control-dams. Consequently, at the end of lactation (day 20) body weight of CR-dams achieved the level of control dams (controls-dams: 291 ± 5 g and CR-dams: 288 ± 4 g).

**Table 2:** Body weight gain and cumulative energy intake during different periods of pregnancy and lactation of dams with free access to standard chow diet (Control-dams) or subjected to 20% calorie restricted diet during the first 12 days of pregnancy (CR-dams).

		**Control-dams**	**CR-dams**
**Pregnancy 1 - 12 days**	Body weight gain (g)	41.0 ± 2.5	11.7 ± 2.1 *
	Cumulative energy intake (kcal)	724 ± 64	561 ± 7 *
**Pregnancy 13 - 20 days**	Body weight gain (g)	70.2 ± 4.7	86.0 ± 5.5
	Cumulative energy intake (kcal)	534 ± 45	604 ± 34
**Lactation 1 - 20 days**	Body weight gain (g)	22.4 ± 1.6	33.1 ± 3.9 *
	Cumulative energy intake (kcal)	3449 ± 77	3348 ± 78

Body weight gain was calculated as the increase of body weight from initial to final weight for each period, and is expressed in grams. Cumulative energy intake during different periods of gestation and lactation was calculated by adding the total amount of food consumed and multiplying by energy density of the diet (3.3 kcal/g), and is expressed in kilocalories. Results are expressed as the mean ± S.E.M. of 7-10 dams per group. Statistics: *, CR-dams different from Control-dams (p < 0.05; Student’s *t*-test).

### Energy intake, anthropometric measurements, circulating parameters and gastric leptin in the offspring

**Figure 1:**
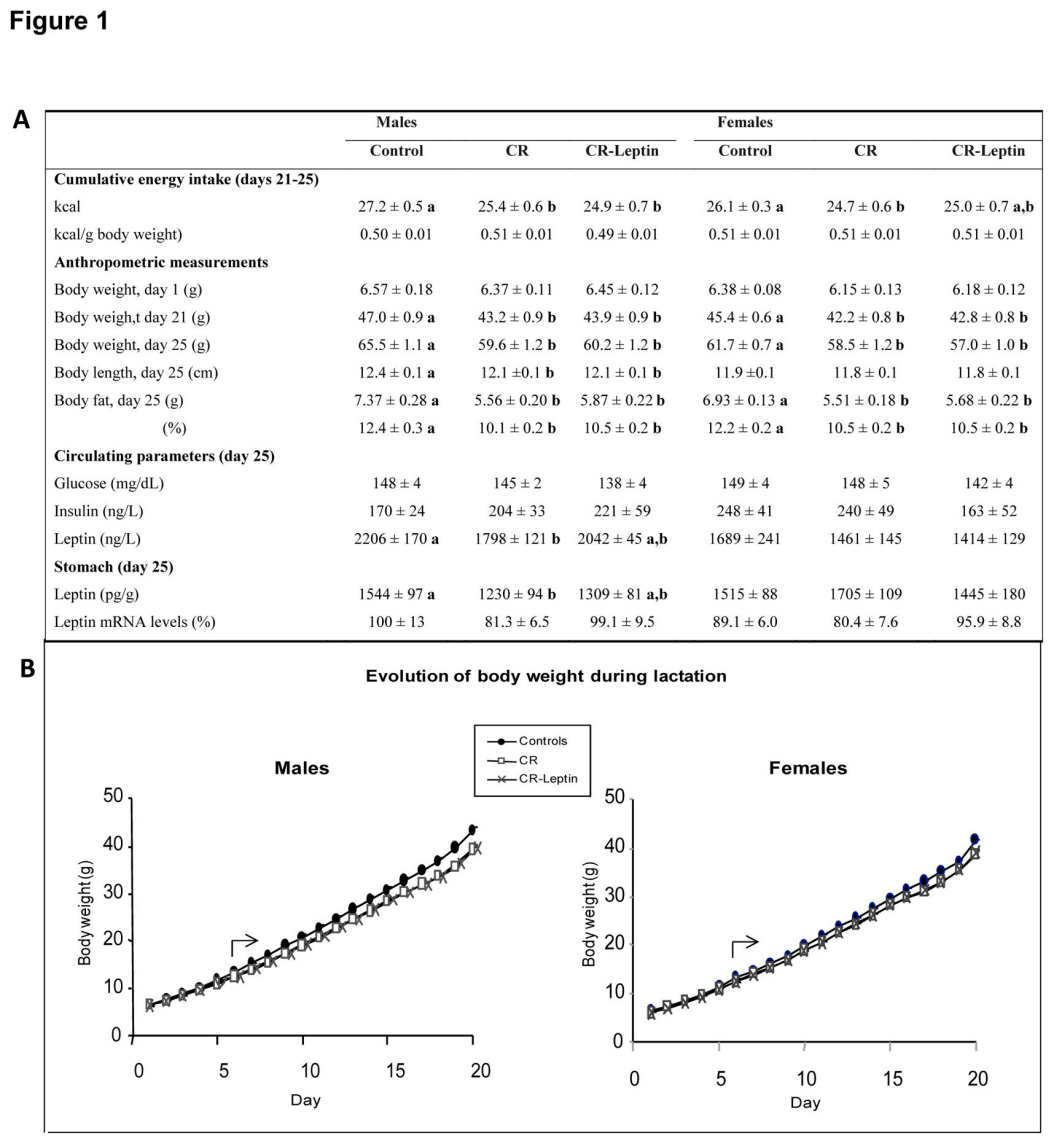
Offspring parameters. A. Energy intake, anthropometric measurements, circulating parameters and leptin mRNA and protein levels in stomach in the offspring of rats with free access to standard chow diet (control), the offspring of 20% calorie restricted dams during the first 12 days of pregnancy (CR), and CR rats daily supplemented with physiological doses of leptin throughout lactation (CR-Leptin). Cumulative energy intake (from postnatal day 21 to 25) was expressed in kcal, and also referred to body weight and expressed in kcal/g. Body weight was measured on postnatal days 1, 21 and 25. The other parameters were determined on day 25. Leptin mRNA levels in stomach were measured by qRT-PCR and expressed as a percentage of the value of control male rats. Leptin levels in stomach were quantified by ELISA and expressed in pg/g tissue. Data are mean ± S.E.M. For cumulative food intake, body weight at different days, body length and body fat content, n = 16-17; for mRNA analysis, n = 10-11; for circulating parameters, n = 6-8. Each group is made up of animals coming from at least six different litters. Statistics: in case of interaction within each sex, data not sharing a common letter (a and b) are significantly different (a≠b) (p < 0.05; LSD *post* *hoc* one-way ANOVA test). B. Evolution of body weight during lactation. The arrow indicates the starting point of significant effects of maternal caloric restriction during gestation on body weight in male and female offspring (CR≠Controls; CR-Leptin≠Controls; p < 0.05; LSD *post* *hoc* one-way ANOVA test).

Data related with energy intake, body weight, body fat and body length measurements, circulating parameters and gastric leptin in the offspring at birth and/or after weaning are summarized in Figures 1A and B. Maternal caloric restriction during gestation did not affect body weight at birth. However, during the suckling period, CR pups gained less weight and exhibited lower body weight that their controls from postnatal day 6 onwards (p < 0.05; LSD *post hoc* one-way ANOVA test). Cumulative energy intake after weaning, from day 21 to day 25, was lower in CR animals with respect to controls (p < 0.05; LSD *post hoc* one-way ANOVA test), although no differences were found when this was referred to body weight. Leptin treatment throughout lactation had no demonstrable effects on body weight of suckling pups, or in post-weaned animals at this juvenile age. However, post-weaned CR-Leptin females showed cumulative food intake slightly higher to that of CR animals, but not different to that of controls (p < 0.05; LSD *post hoc* one-way ANOVA test). At any rate, no differences were found when food intake was referred to body weight.

In addition, CR males, but not females, had shorter body length than their control counterparts, and both CR males and females exhibited lower body fat content than controls, expressed in absolute values, as well as in relative values referred to body weight (p < 0.05; LSD *post hoc* one-way ANOVA test). In turn, leptin treatment throughout lactation had no demonstrable effects on the mentioned parameters (p < 0.05; LSD *post hoc* one-way ANOVA test). 

Analyses of circulating levels of glucose and insulin at postnatal day 25 showed no statistical differences between groups either due to caloric restriction during gestation or due to leptin treatment throughout lactation. However, in males, but not in females, maternal caloric restriction during gestation led to a significant reduction of leptin levels in plasma, which became partially reverted to the levels similar to controls due to leptin treatment throughout lactation (p < 0.05; LSD *post hoc* one-way ANOVA test). Similar trend to that of circulating leptin was found regarding leptin concentration in the stomach (p < 0.05; LSD post hoc one-way ANOVA test). However no significant changes between groups were found concerning gastric leptin mRNA levels. 

### Morphometry and immunohistochemistry of the ARC and PVN hypothalamic nuclei in the offspring

**Figure 2:**
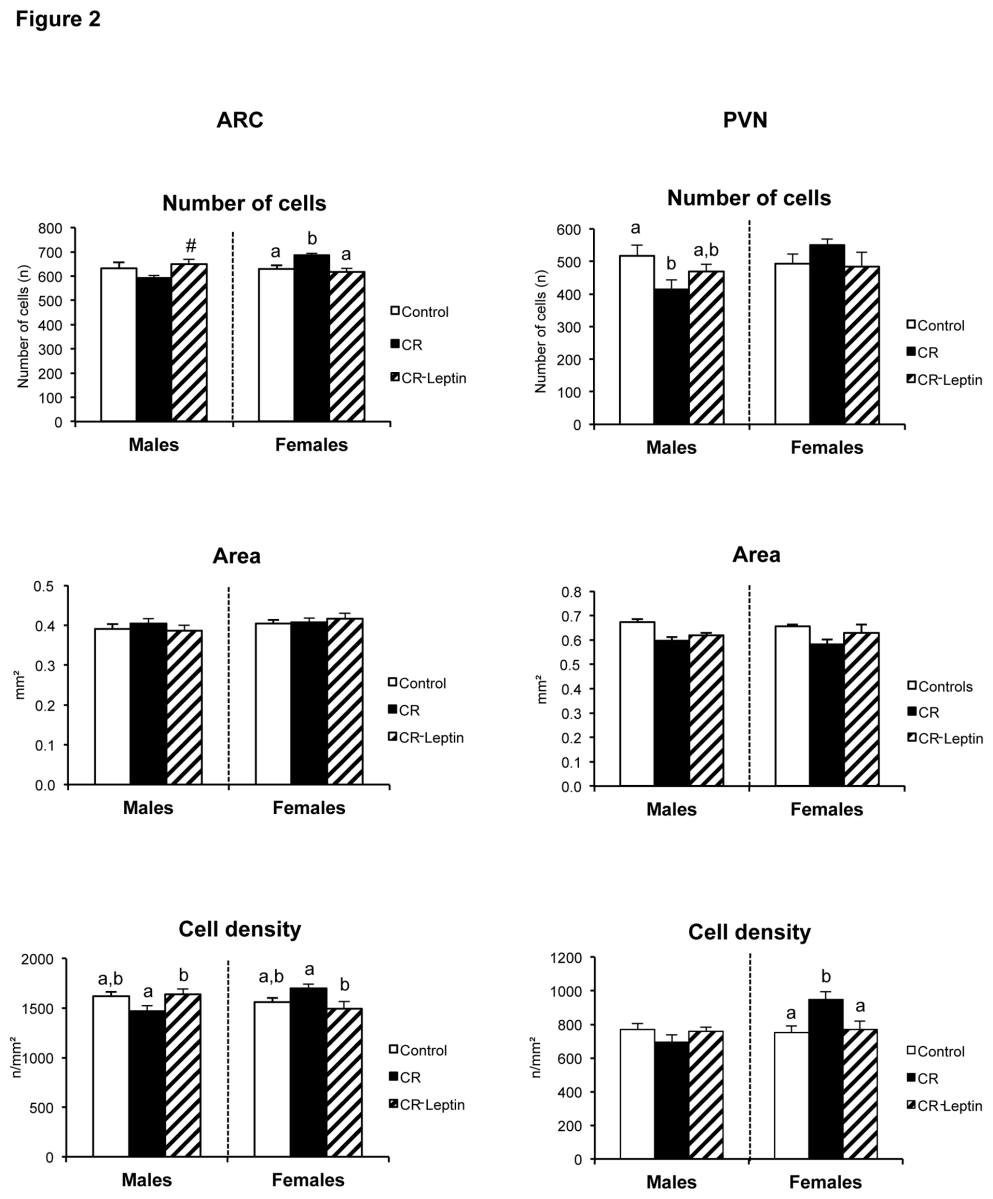
Morphometry of the ARC and PVN hypothalamic nuclei in the offspring. Total number of cells, area and cell density in the arcuate nucleus (ARC) (left panels) and total number of cells, area, and cell density in the paraventicular nucleus (PVN) (right panels) of the hypothalamus of 25-day-old male and female offspring of dams with free access to standard chow diet (controls), the offspring of 20% calorie restricted dams during the first 12 days of pregnancy (CR), and CR rats daily supplemented with physiological doses of leptin throughout lactation (CR-Leptin). The number of cells was determined in hematoxylin/eosin-stained section of the hypothalamus and the area was counted using computerized image analysis software. Neuronal density was calculated dividing total number of cells in nuclei per surface of nucleic area and is expressed as number of cells per square millimeter. Data are mean ± S.E.M. (n = 6-8, coming from at least six different litters). Statistics: ^#^, CR-Leptin different from CR group (p < 0.05; Student’s *t*-test); in case of interaction within each sex, bars not sharing a common letter (a and b) are significantly different (a≠b) (p < 0.05; LSD *post* *hoc* one-way ANOVA test).

Morphometric (Figure 2) studies were performed in ARC and PVN hypothalamic nuclei. Analysis of ARC (Figure 2; left panels) showed that maternal caloric restriction during gestation produced an increase in the total number of hematoxylin/eosin-stained cells in the ARC of CR females compared to their controls (p < 0.05; LSD *post hoc* one-way ANOVA test), but a slight trend toward a decrease in CR males, compared to their controls. Leptin treatment throughout lactation reversed the effects observed in CR females diminishing significantly the total number of cells in ARC relative to their controls (p < 0.05; LSD *post hoc* one-way ANOVA test), as well as leading to the augmentation of their quantity in males (CR-Leptin *vs* CR males; p < 0.05; Student’s *t*-test). A similar tendency was also observed by analyzing cell density. CR-Leptin animals showed the same levels as controls, but different compared to the CR groups (p < 0.05; LSD *post hoc* one-way ANOVA test). No differences were found concerning the area of ARC between experimental groups. 

Morphometric studies in PVN (Figure 2; right panels), in turn, demonstrated that gestational caloric restriction also affected the development of this nucleus, by reducing the total number of cells in males, as well as by increasing their density in females in comparison to their control groups (p < 0.05; LSD *post hoc* one-way ANOVA test). These structural changes in PVN were restored in CR-Leptin animals (p < 0.05; LSD *post hoc* one-way ANOVA test). 

**Figure 3:**
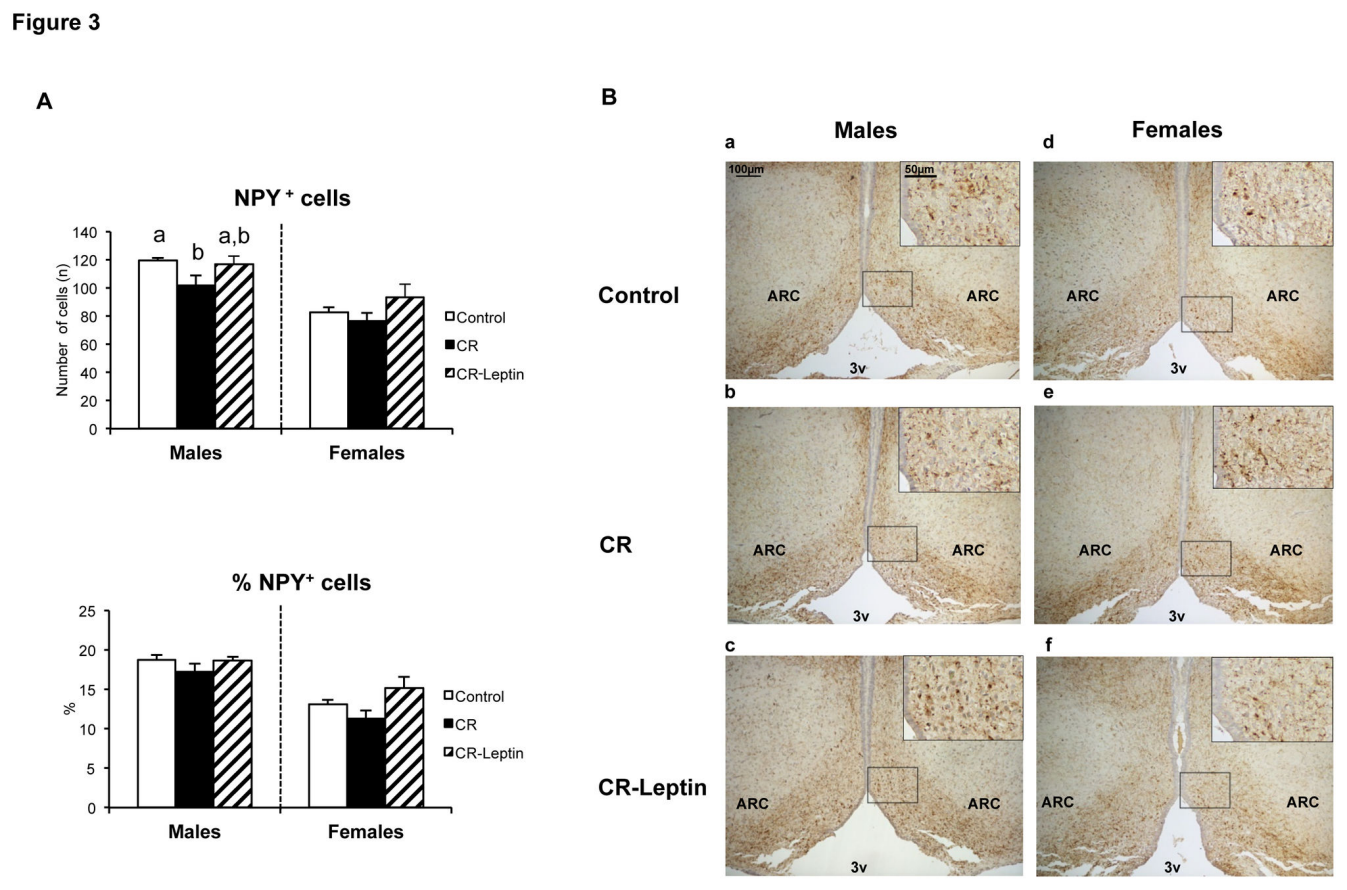
Immunohistochemistry of the ARC hypothalamic nucleus in the offspring. A. Number of NPY positive (NPY^+^) cells and percentage of NPY^+^ cells referred to the total number of cells in the arcuate nucleus (ARC) in the hypothalamus of 25-day-old male and female offspring of dams with free access to standard chow diet (controls), the offspring of 20% calorie restricted dams during the first 12 days of pregnancy (CR), and CR rats daily supplemented with physiological doses of leptin throughout lactation (CR-Leptin). Number of NPY^+^ cells in the ARC was counted using computerized image analysis software, following immunostaining. Data are mean ± S.E.M. (n = 6-8, coming from at least six different litters). Statistics: in case of interaction within each sex, bars not sharing a common letter (a and b) are significantly different (a≠b) (p < 0.05; LSD *post* *hoc* one-way ANOVA test). B. Representative brain sections immunostained for NPY in the arcuate hypothalamic nucleus of the offspring of 25-day-old male (a) and female (d) offspring of dams with free access to standard chow diet (controls), male (b) and female (e) offspring of 20% calorie restricted dams during the first 12 days of pregnancy (CR), and male (c) and female (f) CR rats daily supplemented with physiological doses of leptin throughout lactation (CR-Leptin). Insets: enlargement of the corresponding framed areas. Abbreviations: ARC, arcuate nucleus; 3v, third ventricle. Scale bar: 100 µm and 50 µm for insets.

**Figure 4:**
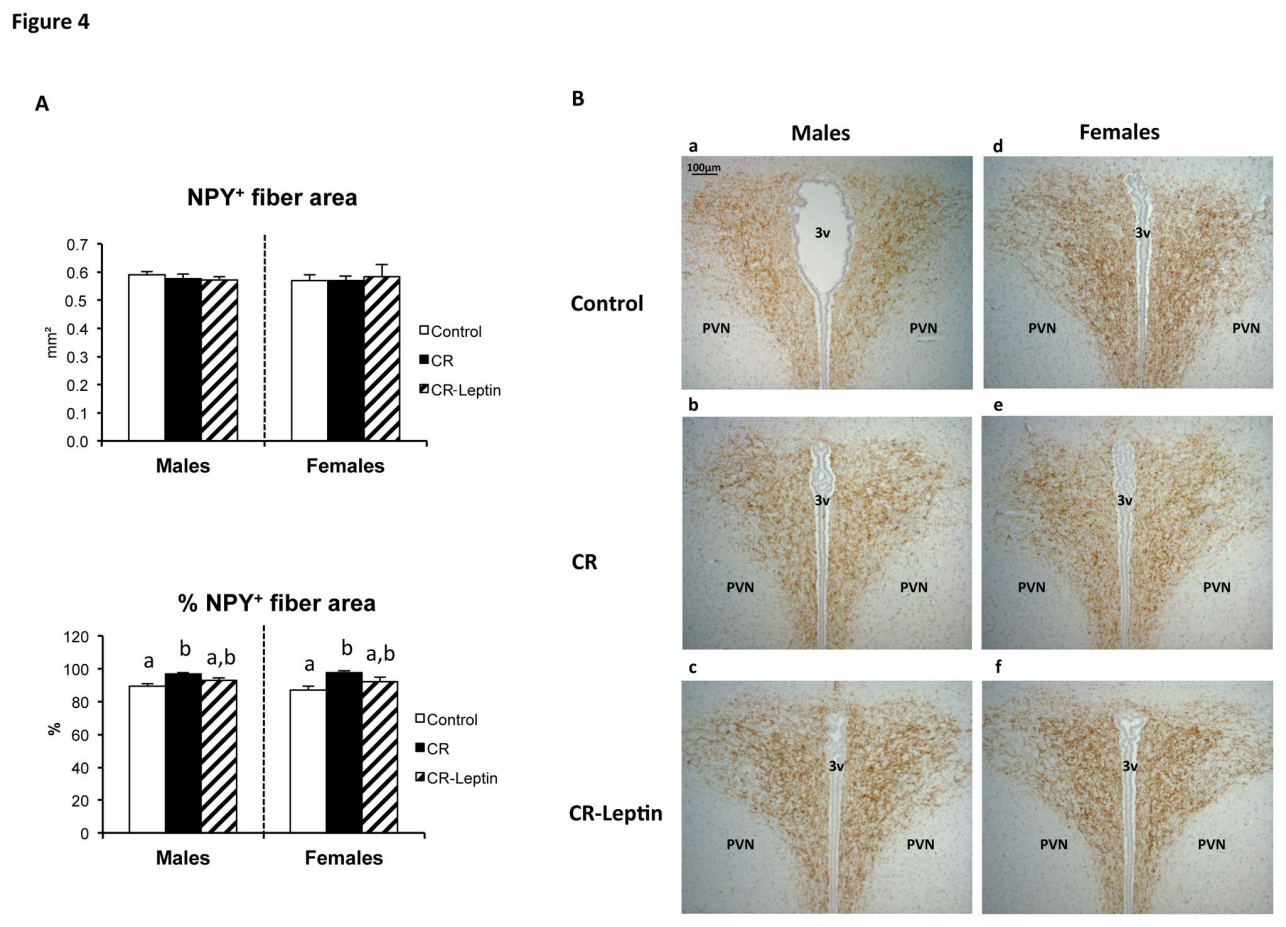
Immunohistochemistry of the PVN hypothalamic nucleus in the offspring. Number of NPY^+^ fiber area and percentage of NPY^+^ fiber area referred to the total area of the paraventicular nucleus (PVN) in the hypothalamus of 25-day-old male and female offspring of dams with free access to standard chow diet (controls), the offspring of 20% calorie restricted dams during the first 12 days of pregnancy (CR), and CR rats daily supplemented with physiological doses of leptin throughout lactation (CR-Leptin). The area of NPY^+^ fibres (expressed in square millimeter) in PVN was estimated using computerized image analysis software, following immunostaining. Data are mean ± S.E.M. (n = 6-8, coming from at least six different litters). Statistics: in case of interaction within each sex, bars not sharing a common letter (a and b) are significantly different (a≠b) (p < 0.05; LSD *post* *hoc* one-way ANOVA test). B. Representative brain sections immunostained for NPY in the paraventicular hypothalamic nucleus of the offspring of 25-day-old male (a) and female (d) offspring of dams with free access to standard chow diet (controls), male (b) and female (e) offspring of 20% calorie restricted dams during the first 12 days of pregnancy (CR), and male (c) and female (f) CR rats daily supplemented with physiological doses of leptin throughout lactation (CR-Leptin). Abbreviations: PVN, paraventicular nucleus; 3v, third ventricle. Scale bar: 100 µm.

In compliance with our previously published results [20], immunohistochemical analysis of NPY^+^ cells (Figure 3) showed that CR males, but not females, presented a significantly lower number of NPY^+^ cells in the ARC nucleus, with respect to their controls (p < 0.05; LSD *post hoc* one-way ANOVA test). The observed diminishment in CR males was partially restored in CR-Leptin males, reaching levels not different to those of controls (p < 0.05; LSD *post hoc* one-way ANOVA test). With respect to the PVN (Figure 4), no significant differences were found in the area of NPY^+^ fibers within experimental groups. However, when this value was expressed relative to the total area of the PVN, both male and female CR animals presented a higher % of NPY^+^ fiber area. Leptin supplementation throughout lactation partially normalized these values to those observed in controls (p < 0.05; LSD *post hoc* one-way ANOVA test).

### mRNA expression levels of AgRP, CART, NPY, ObRb, POMC and SOCS-3 in hypothalamus in the offspring

**Figure 5:**
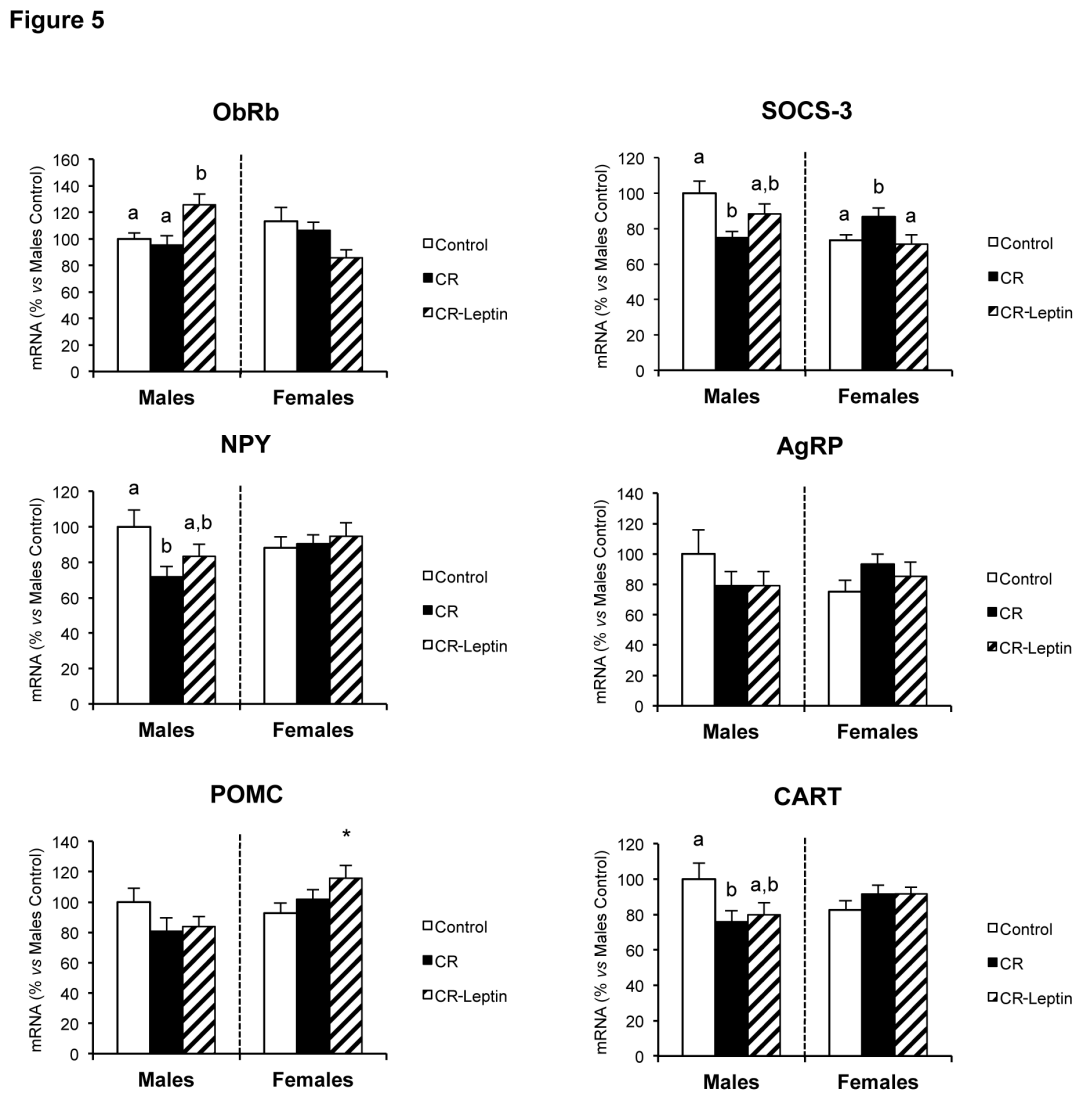
mRNA levels of AgRP, CART, NPY, ObRb, POMC and SOCS-3 in hypothalamus in the offspring. Hypothalamic expression levels of AgRP, CART, NPY, ObRb, POMC and SOCS-3 in 25-day-old male and female offspring of dams with free access to standard chow diet (controls), the offspring of 20% calorie restricted dams during the first 12 days of pregnancy (CR), and CR rats daily supplemented with physiological doses of leptin throughout lactation (CR-Leptin). mRNA levels were measured by qRT-PCR and expressed as a percentage of the value of control male rats. Data are mean ± S.E.M. (n = 10-11, coming from at least six different litters). Statistics: *, CR different from control group (p < 0.05; Student’s *t*-test); in case of interaction within each sex, bars not sharing a common letter (a and b) are significantly different (a≠b) (p < 0.05; LSD *post* *hoc* one-way ANOVA test).

To ascertain whether the apparent changes in hypothalamic structure between groups affected its function, the expression levels of selected hypothalamic genes involved in the control of food intake and energy balance were determined (Figure 5). No statistical differences were observed in the expression levels of InsR either due to gestational caloric restriction or due to leptin treatment throughout lactation (data not shown). Gestational caloric restriction did not affect the expression levels of ObRb either, however, leptin treatment throughout lactation resulted in overexpression of this gene in males, but not in females, with respect to both control and CR rats (p < 0.05; LSD *post hoc* one-way ANOVA test). Regarding SOCS-3 mRNA expression levels were significantly altered in CR animals, with different expression patterns between sexes. In comparison to controls, CR male animals exhibited lower SOCS-3 mRNA levels, whereas in CR females its mRNA levels were increased; interestingly, leptin treatment throughout lactation reversed these effects, resulting in a partial increase of SOCS-3 expression levels in males and a significant downregulation in females (p < 0.05; LSD *post hoc* one-way ANOVA test). In agreement with our previous study in another cohort of animals [20], gestational caloric restriction led to lower expression levels of the orexigenic neuropeptide NPY; however, in the present study, the mentioned effect was observed in males, but not in females (p < 0.05; LSD *post hoc* one-way ANOVA test). In turn, leptin treatment throughout lactation brought about a partial reversion of mRNA levels of NPY in CR-Leptin group of males, which exhibited 16% rise in its expression levels compared to their CR counterparts. No statistical differences between groups were observed in the expression levels of the other orexigenic neuropeptide, AgRP. Concerning anorexigenic neuropeptides, no differences in POMC expression levels were displayed between groups, either due to gestational caloric restriction or due to leptin treatment throughout lactation. However, single comparison between groups revealed that females in CR-Leptin group exhibited a rise in POMC expression levels with respect to female offspring from normally nourished mothers (CR-Leptin *vs* Control females) (p < 0.05; Student’s *t*-test). In accordance with our previous study in another cohort of animals [20], gestational caloric restriction resulted in lower expression levels of another anorexigenic neuropeptide, CART; however, in this study the effect was observed in males, but not in females (p < 0.05; LSD *post hoc* one-way ANOVA test). In turn, leptin treatment throughout lactation brought about a mild reversion of CART mRNA levels, since males in the CR-Leptin group exhibited 6% rise in its expression levels compared with males in the CR group.

## Discussion

We have previously shown that neonate male rats born to perinatally adequately nourished dams orally treated with physiological amounts of leptin throughout the suckling period are protected against the development of obesity and metabolic dysfunction in adulthood [31,32]. Given that the hypothalamic circuitry that programs the capacity to regulate energy homeostasis becomes fully developed during a critical neonatal period, and is particularly susceptible to permanent programming by the early nutritional and hormonal environment [20,23,24,37], here, we investigated the capacity of supplementation with physiological doses of leptin throughout lactation to reverse prior developmental malprogramming induced by maternal undernutrition during gestation. We used a rat model of 20% moderate maternal caloric restriction during the first part of pregnancy. This prenatal condition has been previously described to perturb offspring hypothalamic ARC structure and function [20] and cause dysregulation of appetite in adulthood [15]. This condition was also associated with disruption in the neonatal leptin surge [15]. Interestingly, it was also found in these previous studies that although both genders showed hyperphagia, only males displayed higher body weight in adulthood, particularly under a high-fat diet [6,15].

In the present study, the overall effects of gestational calorie restriction on neuroanatomic and metabolic health outcomes of juvenile rats, particularly in male offspring, were broadly consistent with previously reported results [20]. However, although in our previous study [20] dams restricted calorically showed similar body weight to their controls at the end of the pregnancy, in the present study they exhibited a relatively slower body weight recuperation rate, and despite being fed *ad libitum* with a balanced diet after the restriction period, they managed to completely recover the previous body weight loss only toward the end of the lactation period. We also found here that undernutrition conditions in dams during gestation affected anthropometric parameters of the offspring. Gestational calorie restriction induced slowdown of growth in neonates, which resulted in lower body weight from postnatal day 6, as well as lower body fat mass and length (the latter only in males) compared to their controls (measured at weaning). In addition, food intake in post-weaned rats, from day 21 to day 25, was significantly lower in CR animals compared with controls. Oral supplementation with physiological doses of leptin throughout lactation to the offspring of undernourished dams did not show any apparent effect on the growth of pups during this period. In rats born from adequately nourished dams during gestation, oral supplementation with the same doses of leptin during lactation, as used here, was previously shown to inhibit food intake, however no effect was either found on body weight gain during treatment [31,32]. Vickers et al. observed that daily leptin subcutaneous injection of pharmacological doses (2.5 µg/g/d) from postnatal day 3 to13 resulted in a slowdown of body weight gain during treatment in the offspring of prenatal 30% calorie-restricted dams, which was suggested to be associated with direct effects of leptin on energy expenditure [29,30]. The apparent differences in the effects of leptin during lactation concerning energy balance regulation during the treatment period may be attributed to the administration technique, doses, as well as to the nutritional status of pups. 

Leptin has been shown to play an essential role during critical windows of development in the pathogenesis of programming related disorders [28,38]. Maintaining a critical leptin level during development may allow the normal maturation of tissues and pathways involved in metabolic homeostasis [39]. In normally developing rodents there is an increase in circulating levels of leptin between postnatal days 4-16, the so-called leptin surge [40]. Disruption in leptin surge during perinatal life in rodents has been demonstrated to have lasting consequences by altering the capacity to respond to leptin in adulthood, thus predisposing animals to obesity [38,41]. In fact, we and others have reported that perinatal caloric restriction triggers a reduction or even absence of neonatal leptin surge [15,18], leading to severe alterations in the control of energy balance in adulthood. As a matter of fact, in a previous study applying the same model of gestational caloric restriction, but conducted in another cohort of animals [15], we showed an absence of the leptin surge, occurring in their controls at postnatal day 9. Here, although we did not measure leptin levels during the suckling period, we detected that 25-day-old CR male animals displayed lower circulating leptin levels than their controls. In turn, leptin supplementation throughout lactation normalized their levels to those of controls. This leads us to speculate that the leptin surge was also ameliorated in those animals, and this might be the cue, which brings reversion of altered programming cascade. Similarly, Vickers et al. reported that daily leptin treatment from postnatal day 3 to 13 normalized fasting plasma leptin concentration in female adult offspring of calorie-restricted dams during pregnancy [29]; however, data confirming this normalization during the neonatal and juvenile period were not available in this study. 

The origin of leptin allowing the normalization of its circulating levels in 25-day-old CR-Leptin male animals to those of controls is not known. It does not seem to be directly caused by oral leptin supplementation, since this treatment lasted only until day 21. Moreover, leptin absorption by the stomach in rat pups has been described to be more significant during the first part of the suckling period, but decreases when animals start to eat a solid diet [26]. To ascertain which endogenous supplier was allowing the normalization of circulating leptin concentration in these animals, leptin production was measured in the gonadal adipose tissue depot and in the stomach. Results showed that adipose tissue is unlikely to be responsible because CR-Leptin animals showed no increased fat mass compared with CR animals, nor was higher leptin expression found in this tissue (data not shown). However, although leptin expression levels in the stomach were not significantly different between groups, CR males exhibited lower gastric leptin levels than controls, whereas levels were normalized in CR animals supplemented with leptin during the suckling period, a similar trend to that found for circulating levels. Therefore, it could be speculated that changes in leptin production by the stomach in male animals could contribute to changes in circulating leptin levels, particularly during this period when the transition from milk to solid food occurs and prior to a greater contribution of adipose tissue. It must be highlighted that the alteration in circulating or gastric leptin levels occurring in male offspring as a consequence of gestational maternal caloric restriction was not evident in female animals. No changes were found as a consequence of leptin treatment during lactation either. This sex-dependent difference in leptin profile as a consequence of maternal caloric restriction may account, at least in part, for the different sex-dependent outcomes of this condition in hypothalamus structure and function (described below and summarized in table 3) as well as in adult phenotype, as previously described [15].

**Table 3:** Summary of changes in hypothalamus structure and function.

		**Males**		**Females**	
		**CR vs C**	**CR-Leptin vs C**	**CR vs C**	**CR-Leptin vs C**
ARC	Number of cells	–	–	↑	–
	Area of cells	–	–	–	–
	Cell density	–	–	–	–
	Number of NPY**^*+*^** cells	↓	–	–	–
	% of NPY**^*+*^** cells	–	–	–	–
PVN	Number of cells	↓	–	–	–
	Area of cells	–	–	–	–
	Cell density	–	–	↑	–
	NPY**^*+*^** fiber area	–	–	–	–
	% of NPY**^*+*^** fiber area	↑	–	↑	–
ObRb mRNA levels		–	↑	–	–
SOCS-3 mRNA levels		↓	–	↑	–
NPY mRNA levels		↓	–	–	–
AgRP mRNA levels		–	–	–	–
POMC mRNA levels		–	–	–	↑
CART mRNA levels		↓	–	–	–

Arrows (↑ or ↓) indicates significant changes (increase or decrease, respectively) occurring in the male and female offspring of 20% calorie restricted dams during the first 12 days of pregnancy (CR) vs controls and in CR rats daily supplemented with physiological doses of leptin throughout lactation (CR-Leptin) vs controls; - indicates no significant changes. Notably, changes occurring in CR animals versus controls were not present in CR-Leptin animals versus controls, which is indicative that oral leptin during lactation is able to revert, at least partly, most of the sex-dependent neuroanatomic consequences in the offspring caused by moderate maternal caloric restriction during gestation. See material and methods for statistical details.

In neonate rodents, the neuronal network responsible for food intake and energy balance regulation is progressively established during early postnatal life; in fact, elevated leptin levels during the critical window of development corresponds to the developmental activity of leptin on hypothalamic neuronal circuitry [23]. Morphometric analysis of the hypothalamus in this and other studies have evidenced that gestational caloric restriction affects hypothalamic structure [18–20]. In turn, exogenous leptin administration at pharmacological doses reverses some of the neuroanatomical defects associated with absence or alterations in neonatal leptin surge during the critical window of developmental plasticity [23,24]. Interestingly, here we show that oral supplementation with physiological doses of leptin throughout lactation normalized the total number of cells and cell density in ARC and PVN in the offspring of caloric restricted dams during gestation. Notably, in females, gestational malprogramming did not reduce cell density in PVN, but triggered its increase. In turn, leptin treatment in CR females also brought about its correction. In addition, in agreement with our previous study [20], we found that gestational caloric restriction led to a reduction of immunostained NPY^+^ cells in ARC of CR males, but no changes were found in females. The decrease occurring in CR males was corrected by inducing leptin supplementation throughout lactation. Therefore, according to the neurotrophic action of leptin on the ARC, we could speculate that the restoration of the ARC structure could be a consequence, at least in males, of restored plasma leptin levels. Nevertheless, no obvious difference was observed in the innervations of the PVN by NPY-containing projections, either due to gestational caloric restriction or due to leptin treatment. A similar observation was reported by Delahaye et al., showing that 50% perinatal caloric restriction did not trigger gross abnormalities in the hypothalamic NPY projections from ARC to PVN [18]. However, both male and female CR animals presented a higher relative area of NPY^+^ fibers in the PVN, referred to the total area of this nucleus; this could be tentatively related with a higher predisposition to hyperphagia, as previously described [15]. Interestingly, leptin supplementation throughout lactation partially normalized this ratio to the control values, both in male and female animals. 

The rationale for differences between sexes in the effects of moderate maternal caloric restriction during gestation on hypothalamus structure, particularly affecting total number and density of cells in the ARC and PVN, is not known, but may reflect the interaction between nutritional signals and hormones. As mentioned above, these sex-dependent outcomes may be potentially related with the different effects of this condition on circulating leptin levels, since only males were significantly affected. Notably, it must be pointed out that the detrimental consequences of this condition during prenatal life in adulthood have also been found to be more marked in males than in females [6,11,42]. These results strongly suggest the important role of leptin during a specific window of development to adequately match the physiology of the neonate to its future environment, as previously suggested [43].

We also show here that changes in hypothalamic structure in CR animals were associated with changes in hypothalamic function, affecting expression levels of neuropeptides and factors involved in the regulation of feeding behavior, such as NPY, CART and SOCS-3. Interestingly, oral administration of physiological doses of leptin throughout lactation corrected expression levels of these factors. These adjustments were dependent upon prior developmental programming. Concerning NPY, and similarly to our observation in NPY^+^ immunopositive cells in ARC, the decreased hypothalamic NPY mRNA expression levels occurring in CR male pups were restored in leptin treated animals. Similar effects were observed concerning CART mRNA expression levels. Leptin treatment throughout lactation also reversed programmed altered expression of SOCS-3, with opposite patterns between sexes. The production of SOCS-3 is considered as a marker of functional activation of leptin receptor and intracellular signaling [44]; hence, the restoration of higher levels of SOCS-3 mRNA in CR-Leptin males (similar to controls) could be indicative of a restoration of leptin signaling. However, it is difficult to interpret those changes occurring in females, which are in the opposite direction with respect to males, although they mirror the pattern of changes in total number of cells in ARC and cell density in PVN. On the other hand, although maternal caloric restriction did not influence anorexigenic neuropeptide POMC mRNA levels, it is worth pointing out that CR-Leptin females exhibited relatively higher POMC expression levels with respect to female offspring from normally nourished mothers, what may make them more predisposed for effective ways to control food intake in adulthood. Similarly, hypothalamic ObRb mRNA levels were not significantly affected by maternal caloric restriction during gestation, but CR-Leptin male pups presented higher mRNA expression levels of ObRb than control and CR animals. This suggests that these rats may be more responsive to leptin action, what might confer certain protection against obesity in adulthood. This has not been directly measured here but, leptin supplementation during the suckling period to the offspring of adequately nourished rats, with the same doses as in the present study, was described to improve leptin sensitivity in adulthood [32]. Appetite-related neuropeptides, as well as SOCS-3 and ObRb, have also been reported to be sensitive to leptin treatment in neonates [45]. However, in that study, offspring of adequately nourished dams were intraperitonealy treated with high doses of leptin, thus the interplay between developmental malprogramming and alterations in expression levels of those genes was not addressed. 

In conclusion, we have evidenced that oral supplementation with physiological doses of leptin throughout lactation has the ability to reverse, at least partly, most of the sex-dependent neuroanatomic consequences in the offspring caused by moderate maternal caloric restriction during gestation. This is the first demonstration that a specific compound during lactation may reverse a detrimental trend for obesity acquired by poor nutrition during pregnancy. More concretely, these findings support the relevance of the intake of appropriate doses of leptin throughout lactation, which should be worth considering when searching for strategies to treat and/or prevent development of obesity and its related metabolic disorders starting in the early stages of life.
